# CT Hounsfield units in cervical ossification of the posterior longitudinal ligament-A comparison with other forms of degenerative cervical myelopathy using propensity score matching

**DOI:** 10.1186/s12891-025-09252-0

**Published:** 2025-11-25

**Authors:** Kun Guo, Ying Xia, Yanyan Zong, Yaping Yu

**Affiliations:** Spine Center, Department of Orthopaedics, Changzheng Hospital Naval Medical University (Second Military Medical University), Shanghai, 200003 P. R. China

**Keywords:** X-ray computed tomography, Dual-energy x-ray absorptiometry, Cervical vertebrae, Ossification of the posterior longitudinal ligament, Bone density

## Abstract

**Background:**

Bone mineral density (BMD) constitutes a critical determinant in evaluating instrumentation-related complications among spinal surgery candidates. Recent advancements in diagnostic imaging have established computed tomography (CT) Hounsfield Units (HU) as a reliable biomarker for assessing regional bone mineral density. Nevertheless, only a paucity of studies have explored the association between HU values and patients with cervical ossification of the posterior longitudinal ligament (OPLL).

**Purpose:**

(1) to conduct a quantitative comparison of bone mineral density between cervical OPLL and other forms of degenerative cervical myelopathy (DCM) cohorts by HU values, (2) to contrast the HU values of cervical OPLL among different types and analyze its correlation with DEXA-derived T-scores, (3) to probe into the risk factors for the development of OPLL.

**Materials and methods:**

A total of 376 patients were enrolled, encompassing 168 cases of cervical OPLL and 208 cases of other forms of DCM. We conducted a comparative analysis of bone mineral density (BMD) through measurement of HU values across C1-C7 segments in patients with cervical OPLL versus other forms of DCM patients. Following 1:1 propensity score matching, HU values and T-scores of 124 paired patients were compared between, and the HU values of the four different type of OPLL patients were compared. Secondly, the segmental HU values among OPLL patients and its correlation with T-scores were identified. Lastly, we performed conditional logistic regression to identifythe risk factors for the development of OPLL.

**Results:**

Overall, the cervical OPLL cohort showed higher segmental HU values and DEXA derived T-scores compared with other forms of DCM patients. Before performing a one-way analysis of variance (ANOVA), only C1 HU (*P* = 0.061)and C2 HU (*P* = 0.053༉showed no significant differences among the four different type groups. However, after conducting the ANCOVA test to control for the covariate age, only C5 HU (*P* = 0.005༉and global HU (*P* = 0.025༉showed significant differences. No statistically significant differences were observed among the HU values of C1-C5 (*P* > 0.05). Conversely, the HU values at C6 and C7 were remarkably lower when compared with those of C1-C5 (*P* < 0.0001). The correlation of HU values and T-scores showed a statistically significant positive weak to moderate correlation among all the comparisons (*p* < 0.0001). Conditional logistic regression analysis found that TC ( odds ratio [OR] 2.989, 95% CI 1.979–3.999), UA (OR 1.636, 95% CI 1.427–1.947), Calcium (OR 0.551, 95% CI 0.343–0.884) were significant risk factors for the development of OPLL.

**Conclusions:**

The cervical OPLL patients showed significantly higher segmental HU values and DEXA derived T-scores when compared with the other forms of DCM. In different types of ossification, C5 HU values and global HU values exhibit significant differences. HU values at C6, C7 were significant lower in comparison to C1-C5. Additionally, the level of serum TC, UA and Calcium were significant risk factors for the development of OPLL. This study provides us with a new insight for understanding the bone density of OPLL patients.

## Introduction

Degenerative cervical myelopathy (DCM) is the most common cause of nerve compression and can result in the manifestation of neurological symptoms [1, 2]. DCM includes ossification of the posterior longitudinal ligament (OPLL), disc herniation, subluxation, spondylolishesis and degenerative cervical deformity. OPLL is a specific subtype of DCM. It is defined as the pathological ectopic bone formation of the posterior longitudinal ligament [3, 4]. A recent research indicated that the incidence of cervical OPLL in DCM patients was as high as 18.22% [5]. The pathogenesis of OPLL has not been thoroughly clarified. It is considered to be a multifactorial disorder. Both genetic and environmental factors are thought to play a role in its development, as well as in determining its type and severity [3, 6]. Surgical decompression is an effective procedure to arrest neurological deterioration. A solid fixation after decompression is crucial to the outcomes. Therefore, a precise evaluation of bone quality prior to surgical intervention is essential to ensure favorable operative outcomes.

While dual-energy X-ray absorptiometry (DEXA) remains a cornerstone for bone mineral density (BMD) assessment [7], its technical limitations in spinal evaluation have been well-documented. On the one hand, degenerative spinal alterations, vertebral osteophytes, and vascular calcifications introduce significant interference in lumbar DEXA measurement, resulting in overestimation of true vertebral trabecular BMD [8, 9]. On the other hand, cervical BMD extrapolated from peripheral skeletal sites (lumbar spine or femoral neck) is not accurate and site-specific. A study trying to demonstrate the hypothesis that the bone density of lumbar spine vertebrae is equivalent to its cranial counterparts with quantitative computed tomography (QCT) found that the average BMD of cervical vertebrae was higher (*p* < 0.001) than thoracic and lumbar spine [10]. Over the past years, higher bone BMD have been widely reported and accepted in cervical OPLL as measured by DEXA [11–16]. Meanwhile, a large-scale national cohort study conducted in Korea has revealed a strong correlation between cervical OPLL and osteoporosis [17]. Consequently, the notion that OPLL is linked to higher bone mineral density continues to be a matter of debate.

CT Hounsfield Units (HU) has been widely used in spine surgery to provide overall and localized bone density of the vertebral body by measuring cancellous bone separately [18, 19]. For this purpose, the study sought to perform a retrospective case-control investigation with the aim of conducting a thorough comparative analysis, thereby advancing our comprehension of bone mineral density patterns in patients with cervical OPLL.

## Methods

### Study design

This retrospective cohort study received the approval from the institutional review board of our hospital (LW-KY-2025-042). Due to the retrospective nature of the study, the requirement for informed consent from the subjects was waived. Consecutive patient records from 2020 to 2023 were retrospectively analyzed. Patients were included in this study if they had available preoperative CT scans with three-dimensional reconstruction and dual-energy X-ray absorptiometry (DEXA) bone density examinations conducted within 3 months prior to surgery, encompassing those who underwent spine surgery for DCM [20, 21], including cervical OPLL, disc herniation and hypertrophied ligamentum flavum. A lateral radiograph serves to categorize OPLL into four subtypes, namely continuous, segmental, mixed, and localized [22]. Patients were excluded from the study if they were under 18 years of age, had incomplete preoperative cervical spine CT scans, a history of previous cervical spinal instrumentation, cases that had only undergone decompression surgery, other infections, dialysis patients, rheumatoid arthritis, had tumors or tuberculosis that could potentially affect the reliability of the measurements. Electronic medical records were retrospectively searched to gather demographic data such as age, gender, body mass index (BMI), type of OPLL, laboratory test results including blood Triglyceride (TG), Total Cholesterol (TC), Uric Acid (UA), Calcium and Phosphorus, comorbidities including Hypertension, Diabetes mellitus, Ischemic heart disease, Cerebral stroke and Thyroid disease, medication history, smoking and alcohol consumption history.

### CT Hounsfield units measurement and DEXA parameters

**Fig. 1:**
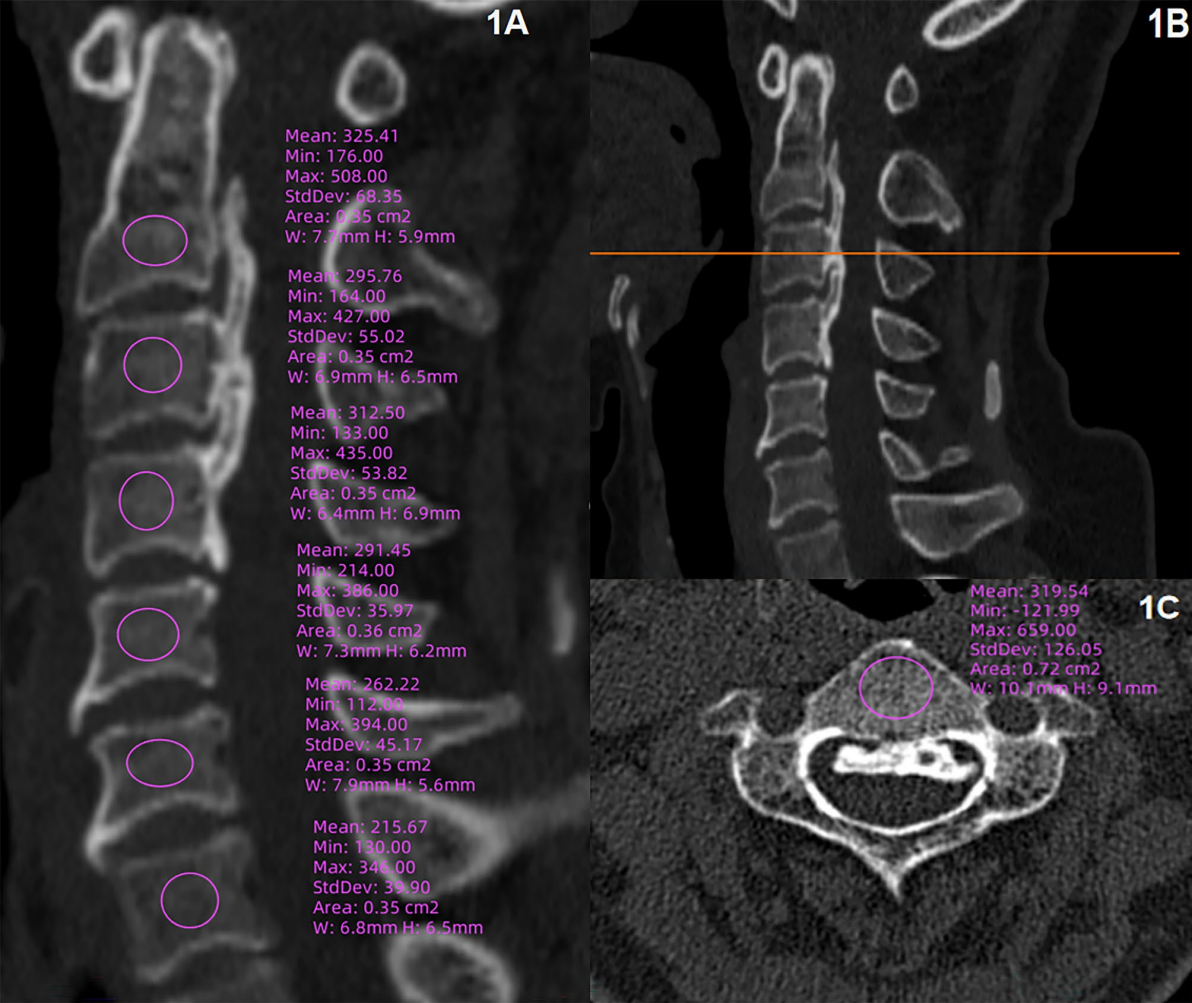
A representative case with cervical OPLL; **1A** The middle-sagittal measurement of C2-C7 HU value; **1B** and **1C**, The position line and middle-axial measurement of HU value

**Fig. 2:**
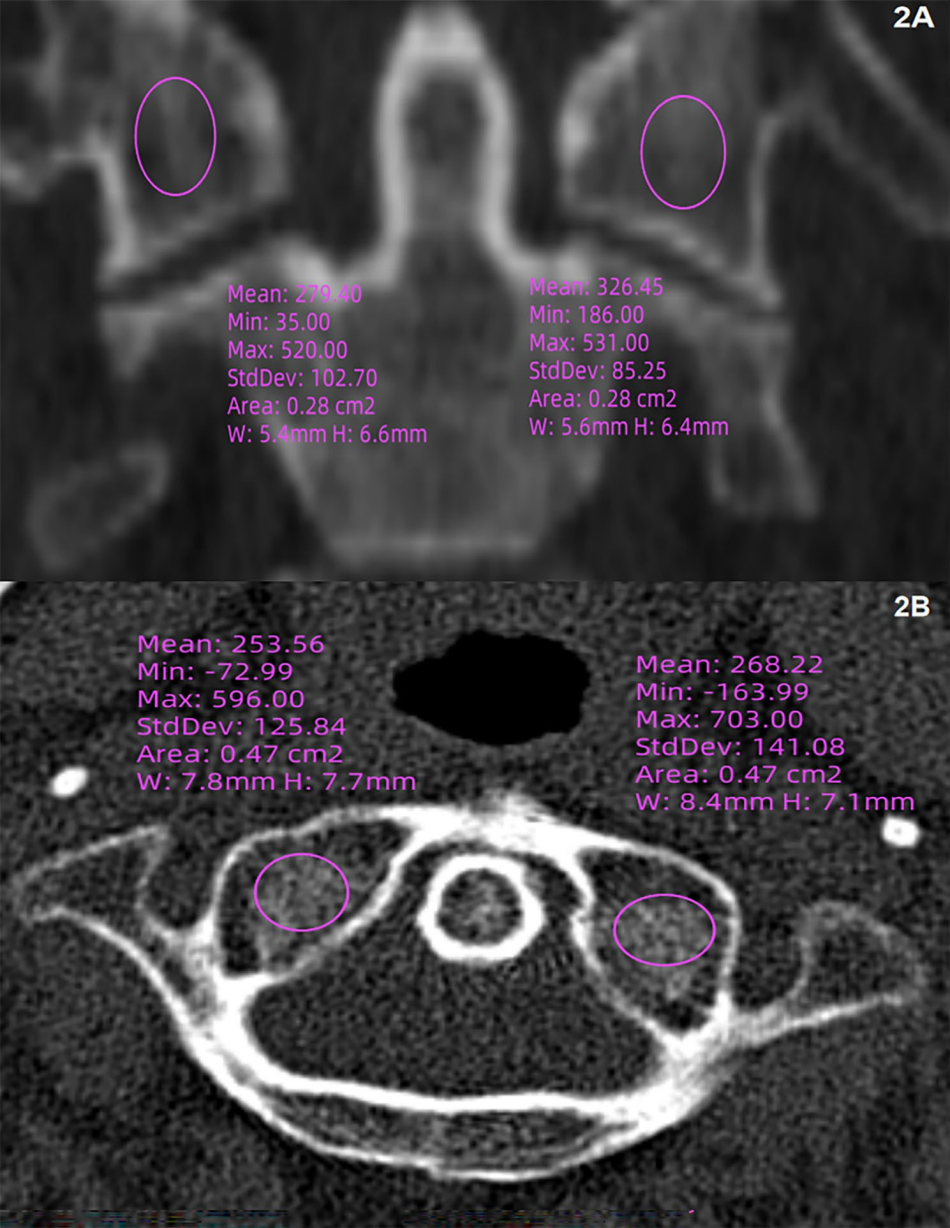
The measurement of C1 HU value; **2A**, the coronal measurement of C1 HU; **2B**, the axial measurement of C1 HU

Multiple methods have been employed to measure bone quality on CT scans through the Picture Archiving and Communication System (PACS). On sagittal views of the vertebral bodies from C2 to C7 (Fig. 1A), a region of interest (ROI) was delineated as described in the literature [9]. This ROI was required to avoid containing the edges of the vertebral body consisting of cortical bone and containing the “vascular zone” or the posterior venous plexus, which is located in the posterior third of the vertebral body [23]. In addition to measuring HU values from sagittal images, measurements were also obtained from axial views. An ROI was drawn on each of the vertebrae from C2 to C7 (Fig. 1A and B). It is also necessary to locate the ROI at the centre of the vertebrae. The measurement of C1 lateral mass was done on coronal and axial view (Fig. 2). A composite HU value of C1-C7 was derived by calculating the mean values, and global HU is the mean HU value of C3–C6. All CT examinations were carried out using a 64 slice dual-source CT scanner (Siemens) with a slice thickness of 1.25 mm with a 0.625-mm interval, and a tube voltage of 120 KV and a tube current of 300 mA. BMD at the lumbar spine and hip was assessed using the GE Lunar iDXA (GE Healthcare, Waukesha, WI, USA).

### Statistical analysis

Statistical analyses were performed using IBM SPSS version 26.0. Patients with cervical OPLL and other forms of DCM were matched with covariates such as age, sex, BMI, comorbidity status, pharmacological exposure history, smoking status and alcohol use history. Propensity score matching was implemented through a non-replacement nearest-neighbor algorithm, employing a caliper width of 0.02 standard deviations for optimal balance attainment. Continuous variables were presented as mean ± standard deviation, and categorical variables are expressed as a percentage. The normality of continuous variables was tested by the Shapiro-Wilk test. For categorical variables, the chi-square test or fisher’s exact test was employed to assess its association, and the Wilcoxon rank-sum test was utilized to analyze differences in matched groups. Student t-test was used to compare the HU values among each vertebral level. To address pairwise comparisons among four different types of cervical OPLL, we conducted additional post - hoc analyses using the Tukey’s Honestly Significant Difference (HSD) test following one-way ANOVA or ANCOVA.​ Spearman’s correlation and the Friedman test were used to evaluate the correlation between HU values and DEXA derived T-scores. The correlation was classified as follows: poor < 0.25, weak between 0.25 and 0.50, moderate between 0.50 and 0.75, and strong > 0.75. To examine the relationship between potential risk factors for developing OPLL, data from matched patients were analyzed using conditional logistic regression. A *p*-value below 0.05 was considered statistically significant.

## Results

A total of 376 patients were evaluated for inclusion, including 168 cervical OPLL patients and 208 other forms of DCM patients. The propensity score matched cohort comprised 124 paired patients for the final analysis. In comparison to other forms of DCM group, the cervical OPLL cohort demonstrated elevated mean BMI values (*p* = 0.0001), younger age distribution (*p* = 0.001), predominant male sex composition (54.03% vs. 58.06%; *p* = 0.013) and higher prevalence of thyroid comorbidities (*p* = 0.044), as detailed in (Table 1). Post-PSM comparative analysis demonstrated successful covariate balance across all predefined matching variables including age, sex, BMI, comorbidities, medication history, smoking and alcohol history between the matched cohorts (Table 2).

**Table 1:** Characteristics of cervical OPLL and other forms of DCM before PSM

	OPLL	Other forms of DCM	SMD	*P* value
	*N*=(168)	*N*=(208)		
Age	58.07±10.85	61.81±9.60	-0.365	0.001†
Male sex, n (%)	103 (61.31)	101 (48.56)	-0.258	0.013†
BMI (kg/m^2^)	25.84±3.66	24.44±3.29	0.466	0.0001†
≤18.49	2 (1.19)	5 (2.40)		
18.5-22.99	29 (17.26 )	67 (32.21 )		
23-27.49	90 (53.57 )	105 (50.48 )		
27.5-32.49	42 (25.01 )	28 (13.46 )		
≥32.5	5 (2.98 )	3 (1.44 )		
Laboratory data				
TG, mg/dl	136.7±42.5	141.7±42.6	-0.024	0.873
TC, mg/dl	200.1±32.3	212.7±33.5	-0.203	0.112
UA, mg/dl	6.63±1.11	6.19±0.94	-0.345	0.007†
Calcium, mg/dl	15.4±0.89	14.9±0.79	-0.368	0.004†
Phosphorus, mg/dl	7.79±0.77	7.85±0.53	-0.307	0.017†
Type of OPLL, n (%)				
Continuous	20 (11.9)			
Segmental	32 (19.1)			
Mixed	55 (32.7)			
Localized	61 (36.3)			
Comorbidity n (%)				
Hypertension, n (%)	50 (29.76 )	74 (35.58 )	-0.124	0.232
Diabetes mellitus, n (%)	21 (12.51 )	28 (13.46 )	-0.047	0.652
Ischemic heart disease, n (%)	4 (2.38 )	13 (6.25 )	-0.191	0.061
Cerebral stroke, n (%)	6 (3.57 )	12 (5.77 )	-0.104	0.311
Thyroid disease, n (%)	9 (5.36 )	3 (1.44 )	0.217	0.044†
Glucocorticoid history, n (%)	5 (2.98 )	9 (4.33 )	-0.072	0.485
Smoking history, n (%)	30 (17.86 )	30 (14.42 )	0.0932	0.372
Alcohol history, n (%)	9 (5.36 )	12 (5.77 )	-0.045	0.663

*PSM* Propensity Score Matching, *DCM* Degenerative Cervical Myelopathy, *BMI* Body Mass Index, *SMD* Standardized Mean Difference

^†^Significant

**Table 2:** Characteristics of cervical OPLL and other forms of DCM after PSM

	OPLL	Other forms of DCM	SMD	*P* value
	*N*=(124)	*N*=(124)		
Age	60.26 ± 10.15	59.66 ± 9.58	0.019	0.882
Male sex, n (%)	67 (54.03)	72 (58.06)	-0.016	0.898
BMI (kg/m2)	24.88 ± 3.07	24.81 ± 3.30	0.012	0.927
≤ 18.49	2 (1.61)	1 (0.08)		
18.5-22.99	29 (23.39)	34 (27.42)		
23-27.49	70 (56.45)	68 (54.84)		
27.5-32.49	20 (16.13)	18 (14.52)		
≥ 32.5	3 (2.41)	3 (2.41)		
Laboratory data				
TG, mg/dl	136.7 ± 42.5	141.7 ± 42.6	0.170	0.455
TC, mg/dl	200.1 ± 32.3	212.7 ± 33.5	-0.022	0.923
UA, mg/dl	6.63 ± 1.11	6.19 ± 0.94	0.056	0.806
Calcium, mg/dl	15.4 ± 0.89	14.9 ± 0.79	0.106	0.643
Phosphorus, mg/dl	7.79 ± 0.77	7.85 ± 0.53	-0.112	0.622
Type of OPLL, n (%)				
Continuous	13 (10.5)			
Segmental	25 (20.2)			
Mixed	37 (29.8)			
Localized	49 (39.5)			
Comorbidity (%)				
Hypertension, n (%)	39 (31.45)	46 (37.10)	0.069	0.590
Diabetes mellitus, n (%)	17 (13.71)	18 (14.52)	-0.023	0.859
Ischemic heart disease, n (%)	4 (3.23)	5 (4.03)	-0.118	0.357
Cerebral stroke, n (%)	4 (3.23)	5 (4.03)	-0.001	1
Thyroid disease, n (%)	4 (3.23)	3 (2.41)	-0.058	0.653
Glucocorticoid history, n (%)	5 (4.03)	4 (3.23)	0.092	0.474
Smoking history, n (%)	20 (16.13)	21 (16.94)	-0.067	0.602
Alcohol consumption, n (%)	6 (4.84)	7 (5.65)	0.043	0.735

*PSM* Propensity Score Matching, *DCM* Degenerative Cervical Myelopathy, *BMI* Body Mass Index, *SMD* Standardized Mean Difference

^†^Significant

### Comparison of bone mineral density and HU values

There were significant differences in DEXA derived bone mineral density between the OPLL and other forms of DCM, with all T values in the OPLL group being higher than those in the other forms of DCM group. There were also significant differences in HU values between the OPLL and other forms of DCM group, and the segmental HU values of the OPLL were all higher than those of other forms of DCM (Table 3).

**Table 3:** Comparison of bone mineral density measured by DEXA and HU value

Bone quality metrics	OPLL *N*=(124)	Other forms of DCM *N*=(124)	*P* value
BMD			
L1 T-score	-0.13 ± 1.74	-0.87 ± 1.36	0.001†
L2 T-score	0.05 ± 1.95	-0.62 ± 1.57	0.004†
L3 T-score	0.87 ± 2.14	0.26 ± 2.65	0.048†
L4 T-score	1.01 ± 2.27	0.57 ± 3.50	0.041†
L1-4 T-score	0.23 ± 1.83	-0.48 ± 1.46	0.001†
Femoral neck T-score	-0.99 ± 1.13	-1.20 ± 1.09	0.043†
Lowest T-score	-1.25 ± 1.28	-1.55 + 1.19	0.03†
CT-HU value			
C1 HU	382 ± 89	360 ± 85	0.039†
C2 HU	354 ± 99	320 ± 87	0.04†
C3 HU	332 ± 90	300 ± 82	*p*<0.001†
C4 HU	339 ± 101	316 ± 92	0.028†
C5 HU	325 ± 88	306 ± 91	0.008†
C6 HU	286 ± 84	271 ± 80	*p*<0.001†
C7 HU	261 ± 71	249 ± 71	*p*<0.001†
Global HU	321 ± 86	298 ± 83	0.04†

*DEXA* dual energy X-ray absorptiometry, *DCM* Degenerative Cervical Myelopathy, Global HU value is the average HU value of C3-C6

^†^Significant

### Comparison of HU values in cervical OPLL among different type

Age showed significant differences among the four different type groups, whereas BMI and gender did not show significant differences. Before performing a one-way analysis of variance (ANOVA), only C1 and C2 HU showed no significant differences among the four groups. However, after conducting the ANCOVA test to control for the covariate age and sex, only C5 HU and global HU showed significant differences (Table 4).

**Table 4:** Comparison of segmental HU values in cervical OPLL among different type

Parameter	Continuous type	Segmental type	Mixed type	Localized type	*p*-Value	ANCOVA adjusted for age and sex *p*-Value
Age (years)	55.5 ± 9.1	59.4 ± 8.3	60.9 ± 11.7	61.8 ± 8.4	0.002†	
Male N %	12 (7.1)	18 (10.7)	35 (20.8)	28 (16.7)	0.156	
BMI (kg/m^2^)	26.9 ± 3.0	25.8 ± 3.1	25.1 ± 3.9	24.0 ± 3.0	0.09	0.536
CT-HU						
C1 HU	344 ± 102	334 ± 72	372 ± 74	318 ± 100	0.061	0.521
C2 HU	377 ± 117	367 ± 101	384 ± 105	333 ± 75	0.053	0.202
C3 HU	351 ± 94	333 ± 80	358 ± 95	310 ± 72	0.036†	0.078
C4 HU	364 ± 96	338 ± 96	372 ± 112	311 ± 69	0.011†	0.096
C5 HU	340 ± 95	327 ± 92	363 ± 88	300 ± 68	0.003†	0.015†
C6 HU	308 ± 86	290 ± 86	320 ± 83	266 ± 71	0.009†	0.125
C7 HU	267 ± 72	272 ± 64	286 ± 67	244 ± 63	0.017†	0.759
Global HU	341 ± 89	322 ± 86	353 ± 88	297 ± 66	0.006†	0.042†

One-way ANCOVA (analysis of covariance) for controlling the covariate age and sex were performed to determine differences among the four groups; Level of significance is set to *p* < 0.05

^†^Significant

### Comparision of segmental HU values and correlation of HU values and T-scores

**Fig. 3:**
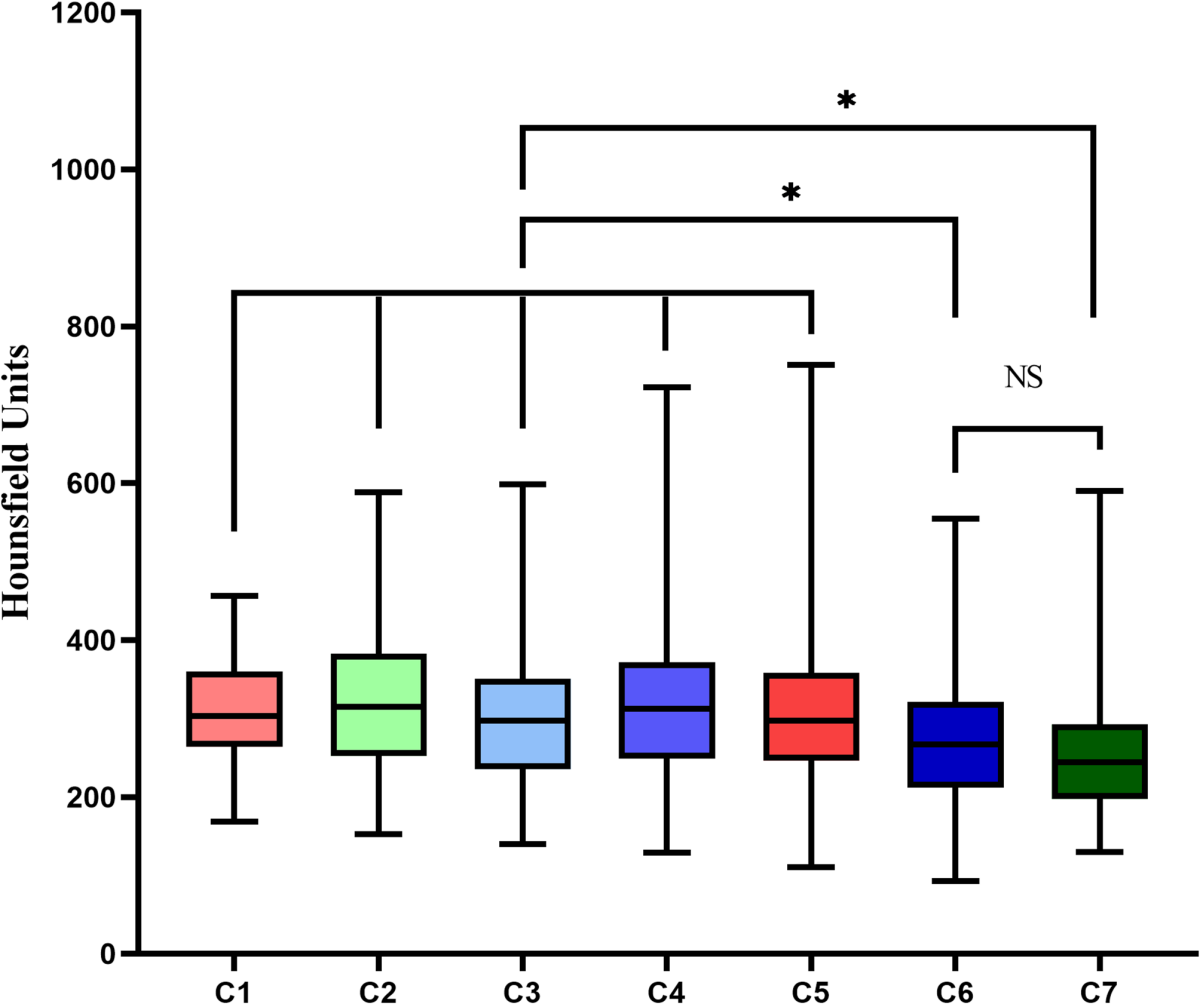
The HU value of C1-C5 segments was greater than that of C6 and C7, but there was no significant difference in the HU value between C6 and C7 segments (*P*>0.05)

**Fig. 4:**
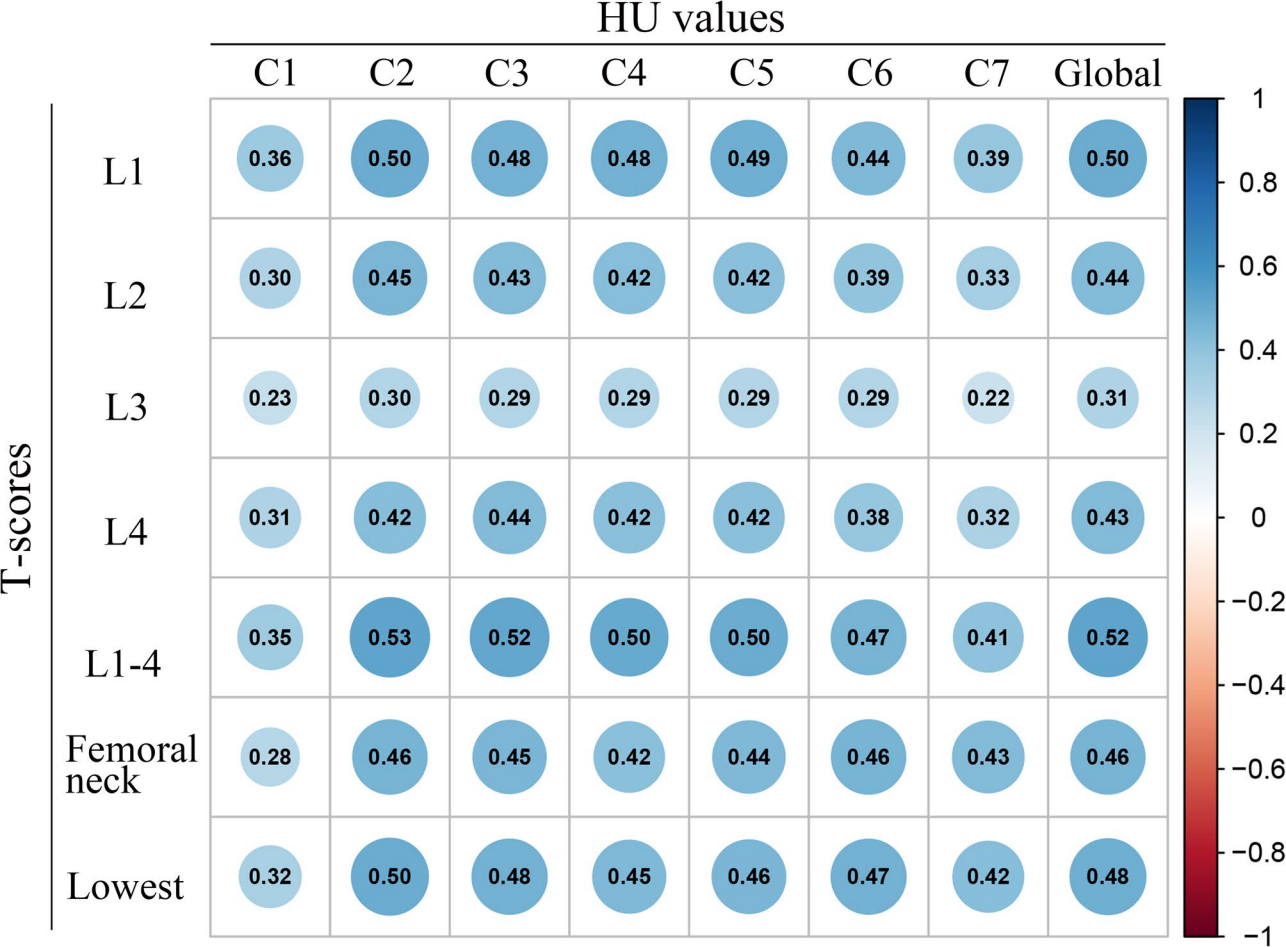
Correlation between HU values obtained from computed tomography and bone mineral density scores obtained from DEXA of the lumbar vertebral bodies and femoral neck. A significant correlations were found among all the comparisons (*p*<0.05)

The mean HU values from C1-C7 was 382, 361, 336, 344, 330, 292, 265 respectively. No statistically differences were found among C1-C5 HU values (*P* > 0.05). HU values at C6, C7 were significantly lower compared with C1-C5 (*P* < 0.0001) (Fig. 3). HU values and T-scores of the lumbar vertebral bodies and femoral neck obtained from DEXA showed a statistically significant positive weak to moderate correlation among all the comparisons (*p* < 0.0001) (Fig. 4).

### Risk factors for the development of OPLL

To elucidate the significant predictors for developing OPLL, we employed conditional logistic regression. The study found that TC (odds ratio [OR] 2.989, 95% CI 1.979–3.999), UA (OR 1.636, 95% CI 1.427–1.947), Calcium (OR 0.551, 95% CI 0.343–0.884) were significant risk factors for the development of OPLL (Table 5).

**Table 5:** Conditional logistic regression analysis of risk factors for developing OPLL

Independent variables	Odds ratio	95% CI	*P* value
Gender	2.827	0.043–183.820	0.626
Age	0.940	0.786–1.123	0.493
BMI	1.444	0.531–3.930	0.071
Global-HU	1.000	1.000–1.000	0.632
TG	1.001	0.992–1.011	0.766
TC	2.989	1.979–3.999	0.034†
UA	1.636	1.427–1.947	0.026†
Calcium	0.551	0.343–0.884	0.014†
Phosphorus	0.646	0.358–1.166	0.147
Hypertension	0.391	0.067–2.273	0.296
Diabetes mellitus	1.483	0.299–7.348	0.629
Ischemic heart disease	0.104	0-139.541	0.537
Cerebral stroke	0.275	0.004–19.074	0.551
Thyroid disease	37.411	0.001-2643995	0.525
Glucocorticoid history	1.312	0.281–6.128	0.730
Smoking history	1.724	0.345–8.618	0.507
Alcohol history	0.356	0.010-12.165	0.566

Global HU value is the average HU value of C3-C6; OPLL, ossification of the posterior longitudinal ligament; BMI, body mass index; Level of significance is set to *p* < 0.05

^†^Significant

## Discussion

To the best of our knowledge, this study revealed trabecular BMD of the cervical OPLL by HU values in patients underwent spine surgery. Existing investigations exploring bone mineral density (BMD) characteristics in cervical OPLL populations have been constrained by critical limitations such as retrospective designs with insufficient sample sizes, inadequate adjustment for established BMD confounders including metabolic comorbidities and pharmacotherapy effects [16]. Consequently, we employed propensity score matching with respect to age, sex, BMI, comorbidities, medication history, smoking and alcohol history. This approach enabled us to compare the HU values and bone mineral density obtained from DEXA between patients with cervical OPLL and those with other forms of DCM patients. Further study was conducted to characterize HU values across various subtypes and to explore the association between segmental HU distributions and risk factors contributing to OPLL development. Overall, after conducting the propensity score matching, cervical OPLL group showed higher HU values and DEXA derived T-scores.

A lower HU values are an indication of poor bone strength and are a potential risk factor for hardware failure and vertebral fracture [24, 25]. The primary findings of our study were that the OPLL group exhibited higher segmental HU values as well as higher global HU values. The comparision of global HU values were slightly differ from that of another study, where they compared the global HU values between the OPLL group and non-OPLL group with PSM analysis [26]. This may be attributed to two primary factors. Firstly, the different calculation methods, one is the average HU values of C2-7 in their study and the other is the average HU values of C3-6 in our study. Secondly, the variation in the disease spectrum among the included participant in the control group could also be one of the contributing factors. However, the global HU values in both studies are generally similar. Meanwhile, another study that compared the segmental vertebral bone quality (VBQ) score reported a lower VBQ score at C3 in cervical OPLL, with no significant differences in other segments compared with the CSM group [27]. The incomplete inconsistency in these findings remains unclear, and further studies are required to assess the practicality of HU values and VBQ scores in evaluating bone density in patients with OPLL. Simultaneously, the DEXA derived T-scores in cervical OPLL group were higher than that of the other forms of DCM group. Our findings align with the outcomes of a prior study [16]. A recent investigation that utilized DEXA to assess the systemic bone mineral density also discovered that patients suffering from cervical OPLL possess a higher whole - body BMD [28].

Traditionally, cervical OPLL is classified into four types [29], and each type necessitates different operative treatment methods. However, there are no studies in which the HU values of the four different types of cervical OPLL are compared. Our study revealed that the segmental HU values exhibited statistically significant differences among the four types, with the exception of C2 HU values. However, after accounting for the age factor, only C5 HU values and the global HU values demonstrated significant differences across the four groups. With increasing age, vertebral bone mass gradually decreases, leading to a reduction in HU values. When age was not controlled for in the one-way analysis of variance (ANOVA), age-related bone mass loss was “superimposed” on the BMD differences associated with different types of cervical OPLL, resulting in the masking of HU value differences in some vertebral segments. ​After controlling for age using analysis of covariance (ANCOVA), the interference of age on BMD was eliminated, and the inherent effect of OPLL type on BMD became prominent. The C5 segment is a focal point of cervical biomechanical stress, and increased stress can lead to an elevation in BMD of the corresponding segment under specific conditions according to Wolff’s law [30]. The C3-C6 segments are the main regions involved in cervical OPLL, and their average HU value is more capable of reflecting the overall ossification degree of OPLL. Further studies are warranted to validate these observations.

Regarding regional bone mineral density (BMD), several studies have tried to evaluate HU values in patients with degenerative cervical spine conditions [31, 32]. Our findings are generally consistent with those of prior research. A retrospective analysis involving 201 patients examined HU values and revealed that these values within specific regions are not integrated but showed considerable variability at each segment. HU values were relatively close from C1-C5, and HU values gradually decreased through T1 which had the minimum average HU [31]. Similarly, in a study of 71 patients underwent ACDF found that the mean trabecular HU value was highest in the mid-cervical spine (C4), decreasing caudally. HUs of C7 and T1 were significantly lower than those of all other levels [32]. In our study, HU values at C6 and C7 were remarkablely lower compared with C1-C5, which is consistent with previous results. Some potential reasons could explain the recurring finding of lower HU values in the lower cervical spine, particularly at the C7 level. One possibility is that C7’s reduced range of motion may result in fewer loading conditions being applied to this vertebra. Additionally, the relatively bigger size of the C7 body compared to the C3-C6 leads to a disproportionate increase in its volume relative to its mass, thereby diminishing the need for higher bone mineral density (BMD) at this location. Lower HU values at C7 could also partially explain some clinically relevant phenomena. Studies on cervical spine fractures have consistently highlighted that the C7 segment accounts for the largest proportion of subaxial vertebral body fractures, followed by C6 and C5, a pattern that may be explained by the lower bone mineral density observed at these levels, as previously documented [33]. Moreover, findings from research on multilevel anterior cervical fusion procedures indicate that construct failure occurs most frequently at the caudal end of the cervical construct, emphasizing the biomechanical vulnerabilities inherent to this anatomical region [34, 35].

Earlier studies have explored risk factors contributing to the development of OPLL in symptomatic patients over time. In this research, we demonstrated that elevated levels of total cholesterol (TC), uric acid (UA), and calcium are associated with the development of OPLL in patients with other forms of DCM, suggesting potential targets for therapeutic intervention. In a study by Doi T et al., higher UA was recognized as the significant predictor of OPLL development in asymptomatic subjects with OPLL [36]. In a recent study by Lu M et al., the development of OPLL was found to be significantly associated with history of calcium supplementation, with regression coefficient of 0.162 (95% confidence interval, 0.010–0.037) [27]. Our findings revealed that increased TC levels constitute a risk factor for OPLL development in patients with other forms of DCM, which are consistent with previous studies [37, 38]. As is widely accepted, the density of vertebral bone declines, the fatty infiltration in trabecular bone rises [39]. The bone marrow microenvironment and osteoblastic activity have been associated with the efficacy of HDL [40]. Yin et al. also discovered that elevated TC levels had the ability to direct the differentiation of marrow stromal cells towards either osteoblasts or bone marrow adipocytes. This process led to changes in bone deposition and contributed to the development of osteoporosis [41]. But these limited studies are not sufficient for us to fully understand the relationship between TC and the development of OPLL, and further clinical studies are required.

### Limitations

This research has multiple limitations. For one thing, since cervical OPLL is a disease of relatively low incidence, the study was carried out at a single center and involved a limited sample. For another, due to certain considerations, we were unable to conduct whole-spine CT scans on the patients in the control group. As a result, we could not entirely eliminate the possibility of having patients with OPLL in the control group. Nevertheless, considering the disease prevalence, we do not think this factor would impact the interpretation of the current findings. This study did not provide a detailed description of whether patients used anti-osteoporotic drugs, such as bisphosphonates and denosumab. Secondly, the study only investigated the glucocorticoid history, but failed to describe the duration and dosage, as well as other drugs that may cause changes in bone mineral density, including antiepileptic drugs, acid-suppressing drugs, and selective serotonin reuptake inhibitors (SSRIs). In addition, baseline data such as the duration of the comorbidity, drug dosage, and the amount of smoking and alcohol consumption were not quantified. The current cross-sectional investigation was primarily designed to examine regional BMD disparities and did not assess clinical outcomes. To fully understand the implications of these BMD differences, prospective longitudinal studies are required to determine their impact on pseudoarthrosis and interbody subsidence, as well as to establish their relevance in clinical practice.

## Conclusions

In conclusion, the cervical OPLL group showed significantly higher segmental HU values and DEXA derived T-scores when compared with the other forms of DCM. In different types of ossification, C5 HU values and global HU values exhibit significant differences. Additionally, HU values at C6, C7 were significant lower compared with C1-C5. Furthermore, elevated serum TC, UA, and calcium levels emerged as notable risk factors associated with OPLL development. This study provided a novel perspective for elucidating bone density characteristics in OPLL patients.

## Data Availability

The datasets used and/or analyzed during the current study are available from the corresponding author on reasonable request.
